# Intermittent theta-burst stimulation with physical exercise improves poststroke motor function: A systemic review and meta-analysis

**DOI:** 10.3389/fneur.2022.964627

**Published:** 2022-08-30

**Authors:** Bixi Gao, Yunjiang Wang, Dingding Zhang, Zongqi Wang, Zhong Wang

**Affiliations:** ^1^Department of Neurosurgery, The First Affiliated Hospital of Soochow University, Suzhou, China; ^2^Institute of Stroke Research, Soochow University, Suzhou, China; ^3^Department of Neurosurgery, Yancheng Third People's Hospital, Yancheng, China; ^4^Department of Anesthesia, The First Affiliated Hospital of Soochow University, Suzhou, China

**Keywords:** intermittent theta-burst stimulation (iTBS), stroke, motor function, Fugl-Meyer assessment (FMA), meta-analysis

## Abstract

**Background:**

Intermittent theta-burst stimulation (iTBS) is an optimized rTMS modality that could modulate the excitability of neural structures. Several studies have been conducted to investigate the efficacy of iTBS in improving the motor function of stroke patients. However, the specific role of iTBS in motor function recovery after stroke is unclear. Hence, in our study, we performed a meta-analysis to investigate the efficacy of iTBS for the motor function improvement of stroke patients.

**Methods:**

MEDLINE, Embase, and Cochrane Library were searched until May 2022 for randomized controlled trials (RCTs).

**Results:**

Thirteen RCTs with 334 patients were finally included in our study. The primary endpoints were the Fugl-Meyer assessment scale (FMA) and Motor Assessment Scale (MAS) change from baseline. We found that iTBS led to a significant reduction in FMA score (*P* = 0.002) but not in MAS score (*P* = 0.24) compared with the sham group. Moreover, standard 600-pulse stimulation showed a better effect on motor function improvement than the sham group (*P* = 0.004), however, 1200-pulse iTBS showed no effect on motor function improvement after stroke (*P* = 0.23). The effect of iTBS for improving motor function only exists in chronic stroke patients (*P* = 0.02) but not in subacute patients (*P* = 0.27).

**Conclusion:**

This study supports that iTBS has good efficacy for improving motor function in stroke patients. Therefore, standard 600-pulse stimulation iTBS therapy is proper management and treatment for chronic stroke.

## Introduction

Stroke has the characteristics of high morbidity, disability rate, and mortality rate on a global scale with the advent of an aging society. According to worldwide research, in 2019, there were 12.2 million new cases of stroke, and 6.55 million people died of stroke events. Stroke accounts for 11.6% of all deaths, which is the second-largest cause of death globally. Besides, stroke is also the third leading cause of disability worldwide (1). Stroke often leads to severe sequelae such as limb hemiplegia, aphasia, spasms, dysphagia, and mood disorders. Early recovery after stroke events is critical to reducing the disability rate (2). The traditional rehabilitation treatments, including physical therapy, speech therapy, hyperbaric oxygen, acupuncture, and massage, were proven to reduce disability after stroke. Besides, new rehabilitation treatments such as non-invasive brain stimulation (NIBS), virtual reality (VR), and rehabilitation robots have been gradually applied in the clinic (3). Among them, NIBS has been proven to affect stroke sequelae in many studies (4–6).

NIBS is essential in neurological disease rehabilitation, promoting neuroplasticity and modulating the excitability of brain structures (7). NIBS include transcranial magnetic stimulation (TMS) and transcranial electrical stimulation (TES) (8). As one of the non-invasive brain stimulation techniques, repetitive transcranial magnetic stimulation (rTMS) offers the opportunity to modulate cortical excitability and correct abnormal cortical activity after stroke, which has been recognized as a promising stroke rehabilitation method (9, 10). Although the exact underlying mechanism remains unclear, it is generally believed that rTMS is effective in improving functional outcomes in patients by modulating motor cortex excitability and inducing neural network reorganization (11). The underlying mechanism of excitatory rTMS can be attributed in part to the removal of magnesium ion blockage in the N-methyl-D-aspartate glutamate receptors during depolarization, which leads to intracellular calcium entry that enhances synapses' post-behavioral learning responses (12). Due to the loss of motor function after stroke, this excitatory stimulation has important implications for relearning during motor rehabilitation (13). In recent years, rTMS has been widely used to treat neuropsychiatric disorders such as depression, epilepsy, pain, and Parkinson's disease (14–17). However, despite the therapeutic potential, many participants reported adverse effects of rTMS, including headache, muscle twitching, and residual hypersensitivity (18, 19).

Intermittent theta-burst stimulation (iTBS) was first used in the human motor cortex by Huang et al. (20) and developed by John Rothwell in his laboratory as an optimized rTMS modality (21). It was first used in the human motor cortex by Huang et al. (20). It presents consistent and durable therapeutic effects in modulating the excitability of neural structures but has many advantages such as low stimulation intensity, short stimulation cycles, and long-term benefits (16, 22). Compared to traditional rTMS methods, one session of iTBS was shorter. Traditional iTBS protocol was standard 600-pulse stimulation, besides, 1200-pulse iTBS were also used in previous studies (23). Briefly, 1200-pulse iTBS were two 600-pulse iTBS with a 15 min interval. A previous study showed that standard 600-pulse iTBS could increase the input-output curve of motor-evoked potentials (IO-MEP). However, 1200-pulse iTBS could attenuate the increase in excitability caused by 600-pulse iTBS (24). For clinical research, several RCTs have been used to evaluate the effects of iTBS on language function, cognitive function, and swallowing function after stroke (25–27). Previous studies have shown that iTBS can reduce aphasia and improve motor function in stroke patients (28, 29). A meta-analysis shows that iTBS has a better effect on the recovery of upper extremity function after stroke than cTBS (30). Compared with cTBS, iTBS exhibits excitatory effects on the cerebral cortex consistent with our need for relearning in post-stroke rehabilitation (13, 24). Besides, there were no pooled analyses of iTBS for post-stroke lower extremity function and balance function. Therefore, we performed a meta-analysis to further explore the effect of iTBS on upper/lower limb motor function and balance function after stroke.

## Methods

### Study protocol

The study was designed and performed following the Cochrane Collaboration format (31).

### Information sources and search strategy

The original materials are systematically retrieved from MEDLINE, EMBASE, and Cochrane libraries. Collect relevant articles published before May 2022. The search terms included “stroke” and “iTBS.” In addition, the list of references included in the trial and meta-analysis was manually screened to avoid omissions. Two researchers (BXG and YJW) examined each article independently to determine whether it met the predetermined inclusion criteria. Any disagreement was settled through another researcher (ZQW) intervening to decide whether to include the study.

### Inclusion and exclusion criteria

The inclusion criteria were: (A) study types: randomized controlled trials; (B) language limitations: English language studies; (C) participants: post-stroke patients; (D) intervention group:iTBS combined with physical exercise; (E) control group: sham iTBS combined with physical exercise; (F) outcome selection: studies aimed at improving motor function.

The exclusion criteria were as follows: (A) study type: protocol, review, comments, meta-analysis, retrospective analysis, case reports, and abstract of the meeting; (B) intervention: non-iTBS intervention; (C) control group: no sham iTBS control group; (D) full-text literature could not be obtained by various methods.

### Study selection and data collection

In this study, two reviewers (BXG and YJW) independently evaluated the literature from the reference list and electronic database according to the above criteria. After two reviewers made a strict selection and evaluation of the literature, the relevant information, including the necessary information of included trials, type of stroke, management of iTBS, adjuvant therapy, and efficacy outcomes, was extracted from the enrolled RCT and summarized in Table 1. The outcomes were the change after the final session from a baseline instead of a follow-up change. For some studies in which SD of change from baseline to endpoint is not given, we roughly estimate the SD value of the data through the following formula.


$$\begin{array}{l} SD_{\text{change}}=\\ \sqrt{S{D^{2}}_{\text{baseline}}+S{D^{2}}_{\text{endpoint}}-\left(2\times Corr\times SD_{\text{baseline}}\times SD_{\text{endpoint}}\right)} \end{array}$$

**Table 1:** Characteristics of the included studies and outcome events.

**References**	**Country**	**Sample size**	**Gender (male/all)**	**Type of stroke**		**Management** **of iTBS**	**Adjuvant therapy**	**Type of motor function**	**Outcomes**
Zhang et al. (32)	Chinese Hong kong	T: cTBS+iTBS (*n* = 14) T: iTBS (*n* = 14) C: sham (*n* = 14)	24/42	24/42 ischemic; 18/42 hemorrhagic	Chronic	1. Type of stimulation: Standard 600-pulse TBS 2. Duration of therapy: 10 session per week, for 3 week 3. Stimulation site: ipsilesional M1	Robot-assisted training after stimulation	Upper extremity motor function	Primary outcomes: 1.FMA-UE; 2.ARAT Secondary outcomes: 1.mean velocity of movement during each session of proximal training; 2.sensorimotor ERD
Xie et al. (33)	China	T: iTBS (*n* = 18) C: sham (*n* = 18)	24/36	20/36 ischemic; 18/36 hemorrhagic	Subacute	1. Type of stimulation: Standard 600-pulse TBS 2. Duration of therapy: 10 consecutive weekdays 3. Stimulation site: contralesional cerebellum	Physical therapy	Lower extremity motor function and balance	Primary outcomes: 1.FMA-LE Secondary outcomes: 1.walking performance; 2.corticospinal excitability
Lin et al. (34)	Chinese Taiwan	T:iTBS (*n* = 10) C:sham (*n* = 10)	17/20	16/20 ischemic; 4/20 hemorrhagic	Chronic	1.Type of stimulation: Standard 1200-pulse TBS 2.Duration of therapy: twice a week for 5 week 3. Stimulation site: leg motor cortex	Physical therapy	Lower extremity motor function	Primary outcome: 1.BBS Secondary outcome: 1.TUG; 2.10-meter walking test; 3. FMA-LE; 4. Barthel Index
Koch et al. (28)	Italy	T:iTBS (*n* = 17) C:sham (*n* = 17)	23/34	100% ischemic	Chronic	1.Type of stimulation: Standard 1200-pulse TBS 2.Duration of therapy: daily for 3 weeks 3.Stimulation site: contralesional cerebellum	Physical therapy	Lower extremity motor function and balance	Primary outcomes: 1.BBS; 2.FMA-LE; 3.Barthel Index
Chen et al. (35)	Chinese Taiwan	T:iTBS (*n* = 11) C:sham (*n* = 11)	14/22	5/22 ischemic; 17/22 hemorrhagic	Chronic	1.Type of stimulation: Standard 600-pulse TBS 2.Duration of therapy: 5 consecutive days per week for 2 weeks 3. Stimulation site: ipsilesional M1	Physical therapy	Upper extremity motor function	Primary outcomes: 1.MAS-UE; 2.FMA-UE; 3.ARAT; 4.BBT; 5.MAL
Watanabe et al. (36)	Japan	T:iTBS (*n* = 8) T: 1Hz stimulation (*n* = 7) C:sham (*n* = 6)	14/21	100% ischemic	Acute	1.Type of stimulation: Standard 600-pulse TBS 2.Duration of therapy:10 consecutive weekdays 3.Stimulation site: ipsilesional M1	Physical therapy and occupational therapy	Upper extremity motor function	Primary outcomes: 1.FMA-UE; 2.SIAS; 3.MAS-UE; Secondary outcomes: 1.grip strength; 2.MEP amplitude
Ackerley et al. (37)	New Zealand	T: iTBS (*n* = 9) C: sham (*n* = 9)	12/18	NM	Chronic	1.Type of stimulation: Standard 600-pulse TBS 2.Duration of therapy:10 consecutive weekdays 3.Stimulation site: ipsilesional M1	Physical therapy	Upper extremity motor function	Primary outcomes: 1.ARAT; 2. FMA-UE
Hsu et al. (38)	Chinese Taiwan	T: ITBS (*n* = 6) C: Sham (*n* = 6)	8/12	100% ischemic	Subacute	1.Type of stimulation: Standard 1200-pulse TBS 2.Duration of therapy:10 consecutive weekdays 3.Stimulation site: ipsilesional M1	Medical and rehabilitation treatments	Upper extremity motor function	Primary outcomes: safety and tolerability; Secondary outcomes:NIHSS, mRS, UE-FMT, ARAT, and affected aMT and MEPs from ECR
Sung et al. (39)	Chinese Taiwan	T:1 Hz rTMS+iTBS (*n* = 15) T: Sham rTMS +iTBS (*n* = 12) T:1 Hz rTMS+ShamiTBS (*n* = 13) C:Sham (*n* = 14)	41/54	NM	Chronic	1.Type of stimulation: Standard 600-pulse TBS 2.Duration of therapy:5 days a week for 4 weeks 3.Stimulation site: ipsilesional M1	Physical therapy	Upper extremity motor function	1.WMFT; 2. FMA; 3. MRC; 4. RT; 5. FT;
Lai et al. (40)	Chinese Taiwan	T(*n* = 21):ITBS. T(*n* = 17):ITBS. T(*n* = 17): ITBS C(*n* = 17): SHAM	41/72	100% ischemic	Chronic	1.Type of stimulation: Standard 600-pulse TBS 2.Duration of therapy:10 consecutive weekdays 3.Stimulation site: FDI hot spot	Physical therapy	Hand movement function	Cortical excitability: MEP, motor map area Motor function assessments: WFMT), Functional Ability Scale, RT, FT
Chen et al. (41)	China	T: iTBS (*n* = 16) C: sham (*n* = 16)	32/78	18/56 ischemic; 14/44 hemorrhagic	Subacute	1.Type of stimulation: Standard 600-pulse TBS 2.Duration of therapy:5 days/week for 2 consecutive weeks 3.Stimulation site: ipsilesional lateral cerebellum	Conventional physical therapy	Upper extremity motor function	Primary Outcomes: 1. MAS; 2.MTS; 3.SWV Secondary Outcomes: 1. Barthel Index; 2. Hmax/ Mmax ratio
Chen et al. (42)	China	T: iTBS+VCT (*n* = 12) C: sham+VC (*n* = 11)	18/78	8/34 ischemic; 15/66 hemorrhagic	NM	1.Type of stimulation: Standard 1,200-pulse TBS 2.Duration of therapy:15 consecutive working days (3 weeks) 3.Stimulation site:the hand motor area of the affected hemisphere	Virtual reality-based cycling training	Upper extremity motor function	Primary outcomes: 1.FMA-UE; 2.MAS-UE Secondary outcomes: 1.ARAT; 2.BBT; 3.NHPT; 4.MAL
Liao et al. (43)	China	T: iTBS (*n* = 15) C: sham (*n* = 15)	21/70	15/50 ischemic; 15/50 hemorrhagic	Subacute and chronic	1.Type of stimulation: Standard 600-pulse TBS 2.Duration of therapy: 5 times per week (Monday to Friday) for 2 weeks in total. 3.Stimulation site:contralesional cerebellum	Physical therapy	Lower extremity motor function	Primary Outcomes: 1. BBS; 2.FMA-LE; 3. Barthel index.

Acute stroke means stroke <1 month. Subacute stroke means stroke from 1 to 6 months. Chronic stroke means stroke more than 6 months. NM means not mentioned.

### Outcome measures

The primary outcomes included the Fugl-Meyer assessment scale (FMA) and Motor Assessment Scale (MAS). The secondary outcomes included the Action Research Arm Test (ARAT), Wolf Motor Function Test (WMFT), Berg balance scale (BBS), and Barthel Index (BI). The most commonly used clinical evaluation of motor function is scale evaluation. FMA is now widely used in the clinical evaluation of motor function, which has good consistency, responsiveness, and accuracy, while MAS can better supplement the shortcomings of hemiplegic limb function scales such as the FMA scale. Besides, FMA is the most widely used in the assessment of spasticity and motor function, MAS is a functionally oriented assessment scale suitable for clinical use. Therefore, FMA and MAS were performed as primary outcomes. ARAT and WMFT were mainly used to evaluate the function of the upper limb and performed in our study to comprehensively evaluate the improvement of upper limb function. BBS was mainly used to evaluate the balance function. BI is mainly used to determine the activities of daily living. The ultimate goal of rehabilitation research is to enable patients to live partially independent lives, therefore we used the BI index in this study. We also performed subgroup analyses, including upper and lower extremities, stimulation intensity, and stroke stages.

### Quality evaluation of included studies

The Cochrane bias risk assessment tool is used to assess the risk of bias in the included RCTs, including selection (including random sequence generation and allocation concealment), implementation (including blind evaluation of researchers and subjects), measurement (blind evaluation of study outcomes), follow-up (the integrity of outcome data), reporting (selective reporting of findings) and other (other sources of bias). The risk of bias can be divided into high, uncertain, or low. PEDro scale was also used in the quality assessment of included RCTs from the perspective of rehabilitation medicine (44).

### Summary measures and synthesis of results

Revman (version 5.3) software was used to analyze the extracted data. Mean difference [MD]; 95% confidence interval [CI] were analyzed using continuous outcomes, respectively, and were calculated using a random-effect model. The random-effect model can effectively avoid bias due to the large differences in the results of the included studies, different population distributions, and different iTBS regimens. I^2^ analysis was used to assess the degree of heterogeneity between studies: I^2^ < 30% represents “low heterogeneity,” 30% < I^2^ < 50% means “moderate heterogeneity” and I^2^ > 50% means “substantial heterogeneity.” *P* < 0.05 was considered significant, and a two-tailed test was used in all analyses.

## Results

### Search results

**Figure 1:**
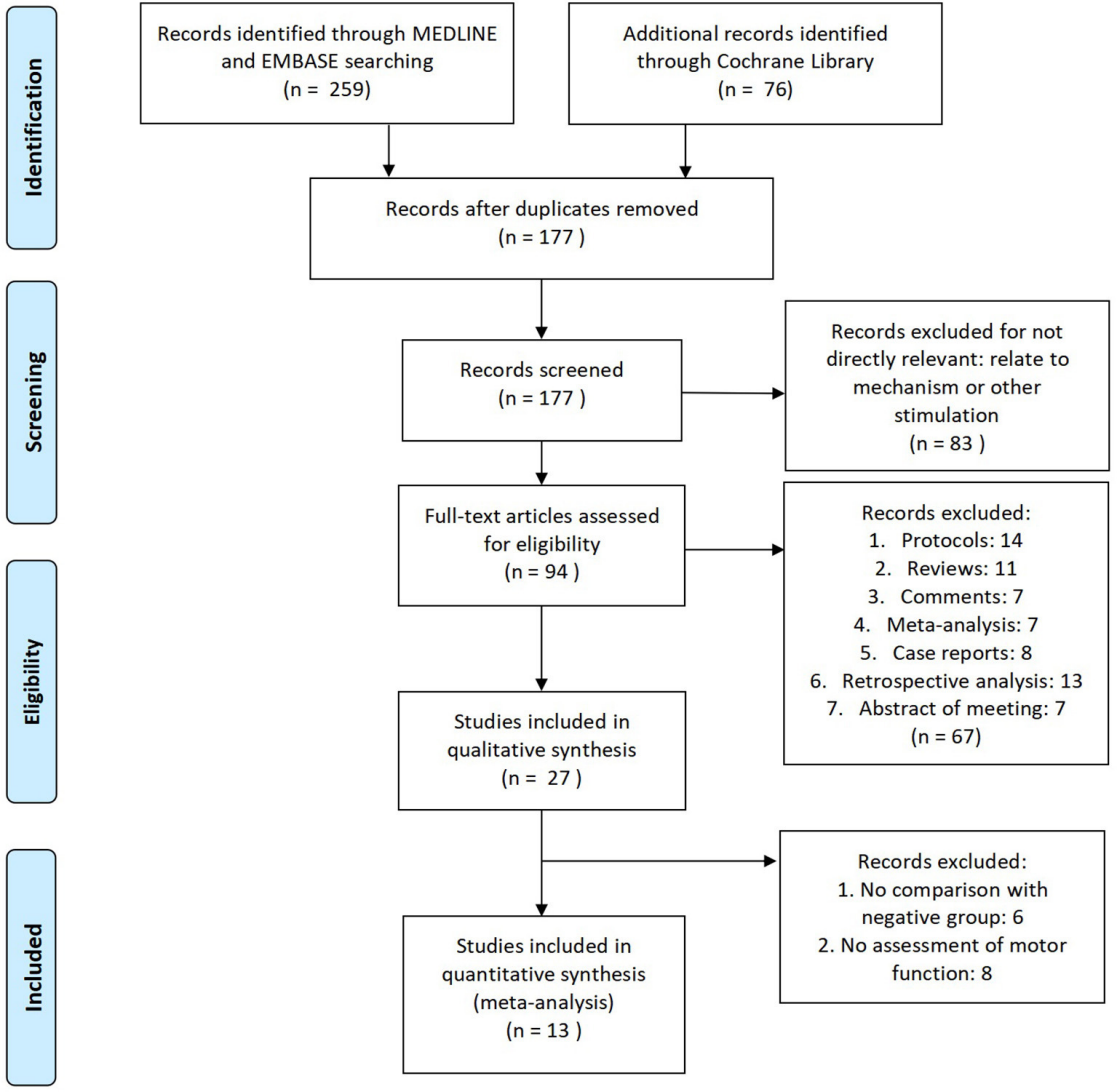
The study search, selection, and inclusion process.

We identified 259 titles and abstracts through MEDLINE, EMBASE, and 76 through Cochrane Library (Figure 1). After removing the duplicated records, 177 full-text articles were evaluated for eligibility. Among them, 83 studies were not directly relevant because of being related to the mechanism or other stimulation. In the remaining articles, 67 articles were excluded because of the restriction of publication types: protocols, reviews, comments, meta-analysis, case reports, retrospective analysis, and abstract of meeting. Besides, among the articles on qualitative synthesis, there were six studies without comparison with the negative group and eight studies without assessment of motor function. Eventually, 13 RCTs containing 334 patients (iTBS, *n* = 170; Sham, *n* = 164) were included in quantitative synthesis (28, 32–43). The main characteristics of 13 RCTs are listed in Table 1.

### Primary outcomes

**Figure 2:**
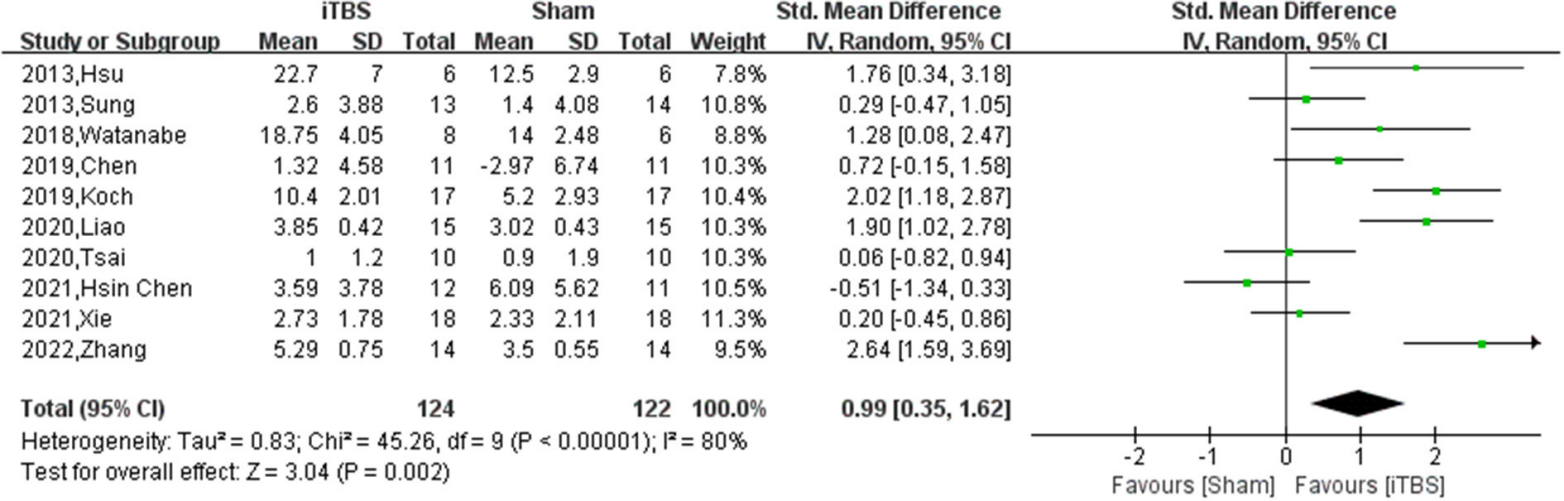
The pooled Std.MD of FMA change from baseline. The green square indicates the estimated Std.MD for each RCT. The size of the green square indicates the estimated weight of each RCT, and the extending lines indicate the estimated 95% CI of Std.MD for each RCT. The black diamond indicates the estimated Std.MD (95% CI) for all patients.

**Figure 3:**
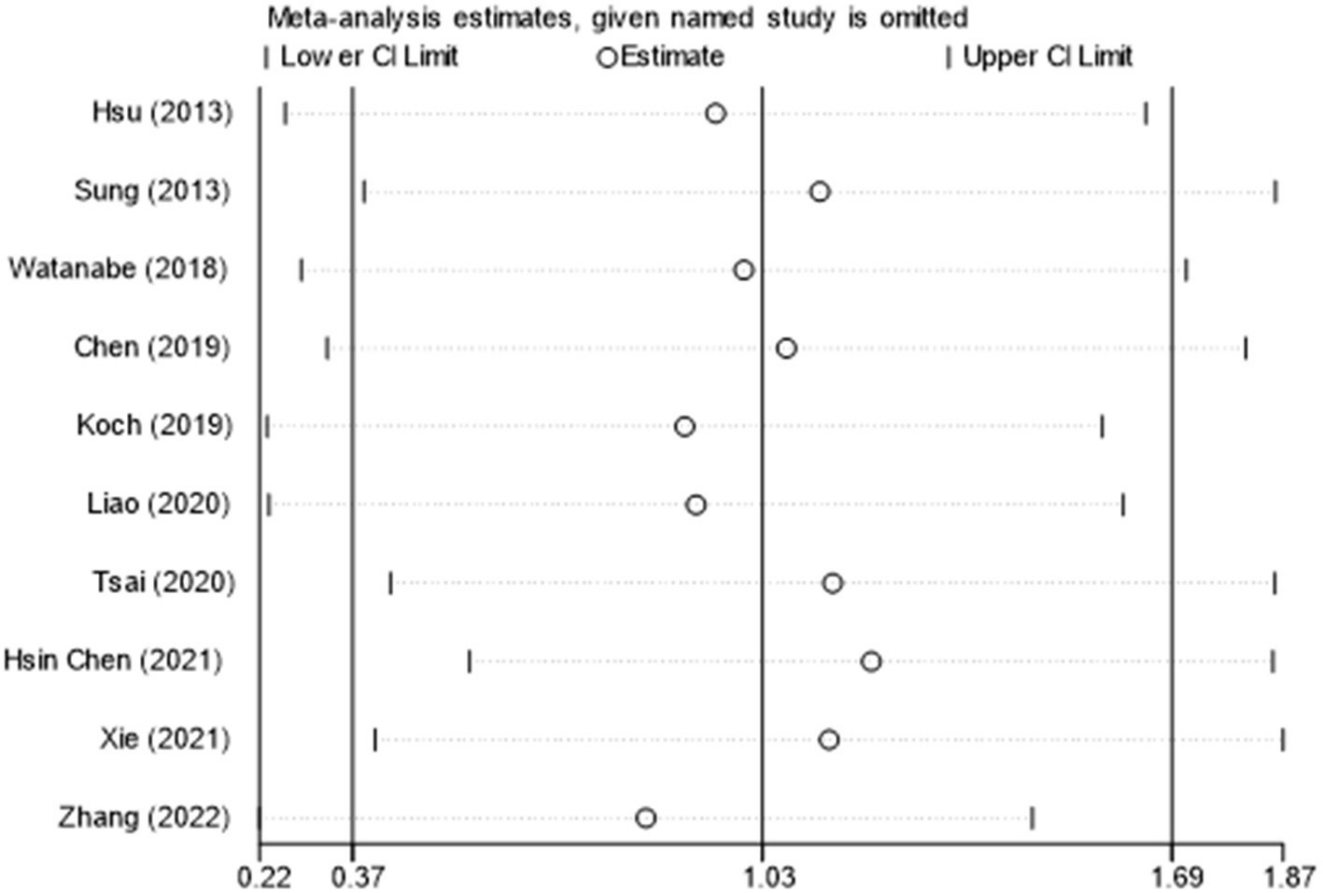
The sensitive analysis of FMA change from baseline. The white circle indicates the pooled Std.MD for excluding each RCT. The extending lines indicated the pooled 95% CI of Std.MD for excluding each RCT.

**Figure 4:**
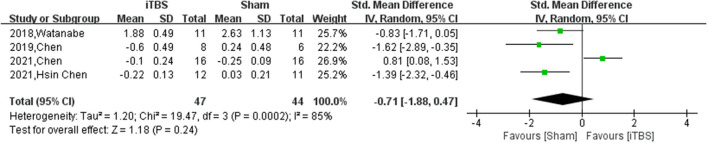
The pooled Std.MD of MAS change from baseline. The green square indicates the estimated Std.MD for each RCT. The size of the green square indicates the estimated weight of each RCT, and the extending lines indicate the estimated 95% CI of Std.MD for each RCT. The black diamond indicates the estimated Std.MD (95% CI) for all patients.

Ten of thirteen RCTs enrolling 246 patients were pooled to analyze the FMA change from baseline in the iTBS and the sham iTBS group. Patients in the iTBS group had a significantly increased FMA score compared with the sham iTBS group (Study number = 10, Std.MD = 0.99 95% CI = 0.35–1.62, *P* = 0.002; Figure 2). The heterogeneity of analysis of the FMA score was 80% (Figure 2). Sensitive analysis and subgroup analysis were implemented to assess the source of heterogeneity and the effect of iTBS in different populations. Sensitivity analysis of overall FMA change from baseline showed that all of the consolidated results were stable (Figure 3). Besides, we divided the included studies into different subgroups according to the type of motor function, stroke, and stimulation (Table 2). When assessing the type of motor function, iTBS could significantly increased the FMA score no matter in upper extremity (Study number = 6, Std.MD = 0.97 95% CI = 0.07–1.97, *P* = 0.04; Table 2) and lower extremity (Study number = 4, Std.MD = 1.03 95% CI = 0.00–2.06, *P* = 0.05; Table 2). However, iTBS showed no benefit in FMA change in subacute stroke (Study number = 2, Std.MD = 0.85 95% CI = −0.66–2.35, *P* = 0.27; Table 2) compared with chronic stroke (Study number = 5, Std.MD = 1.12 95% CI = 0.18–2.06, *P* = 0.02; Table 2). Considering the different regimens of iTBS in the included studies, we performed a subgroup analysis on the type of stimulation. The results showed that standard 600-pulse stimulation had a better effect on increasing FMA score than the sham iTBS group (Study number = 6, Std.MD = 1.12 95% CI = 0.36–1.88, P = 0.004; Table 2). However, standard 1200-pulse stimulation showed no effect on the FMA score (Study number = 4, Std.MD = 0.79 95% CI = −0.49–2.07, *P* = 0.23; Table 2). Besides, 4 of 13 RCTs enrolling 91 patients were pooled to analyze the MAS change from baseline in the iTBS group and the sham group. ITBS could not significantly increase MAS score compared with the sham group (Study number = 4, Std.MD = −0.71 95% CI = −1.88–0.47, *P* = 0.24; Figure 4). Sensitivity analysis of overall MAS change from baseline showed that all consolidated results were stable (Supplementary Figure S1).

**Table 2:** Subgroup Analysis of iTBS for FMA change from baseline.

	**No. of studies**	**The change of FMA from baseline**	
		**Std. mean difference, 95% CI**	***P* value**
1. **Type of motor function**
Upper	6	0.97 (0.07, 1.87)	0.04
Lower	4	1.03 (0.00, 2.06)	0.05
2.**Type of stroke**
Subacute	2	0.85 (−0.66, 2.35)	0.27
Chronic	5	1.12 (0.18, 2.06)	0.02
3. **Type of stimulation**
Standard 600-pulse	6	1.12 (0.36, 1.88)	0.004
Standard 1,200-pulse	4	0.79 (−0.49, 2.07)	0.23

### Secondary outcomes

The secondary outcomes are divided into two types: further assessment of motor function and the improvement of life quality. We assessed the ARAT, WMFT, and BBS in the first type. The results showed that iTBS could increase the ARAT score compared with the sham group (Study number = 5, Std.MD = 2.28 95% CI = 0.83–3.73, *P* = 0.002; Table 3). Nonetheless, iTBS showed no benefit in balance improvement compared with the sham group (Study number = 2, Std.MD = 0.32 95% CI = −0.24–0.88, *P* = 0.26; Table 3). When assessing the WMFT change from baseline, iTBS showed no significant benefit in improving upper limb motor function (Study number = 2, Std.MD = 2.11 95% CI = −0.59–4.80, *P* = 0.13; Table 3). However, there was considerable heterogeneity between the two included studies. The results showed a trend of increased WMFT score, which could seem like a generation of a hypothesis. In another type, though iTBS could improve motor function, its effect on improving life quality was not significant (Study number = 3, Std.MD = −0.04 95% CI = −0.39–0.48, *P* = 0.84; Table 3). The forest plots and sensitive analyses of secondary outcomes were performed in Supplementary Figures S2–S11.

**Table 3:** Secondary outcomes of iTBS for stroke.

**Outcomes**	**No. of studies**	**Std.mean difference**	**95% CI**	**I^2(^%)**	***P* value**
ARAT*	5	2.28	0.83–3.73	87	0.002
WMFT^#^	2	2.11	−0.59–4.80	94	0.13
BBS^&^	2	0.32	−0.24–0.88	0	0.26
BI^&^	3	−0.04	−0.39–0.48	3	0.84

^*^Sensitivity analysis showed that all of the consolidated results were stable.

^#^Sensitivity analysis showed that there was considerable heterogeneity between the two included studies.

^&^Results were performed after excluding highly sensitive trial.

### Risk of bias in included studies

**Figure 5:**
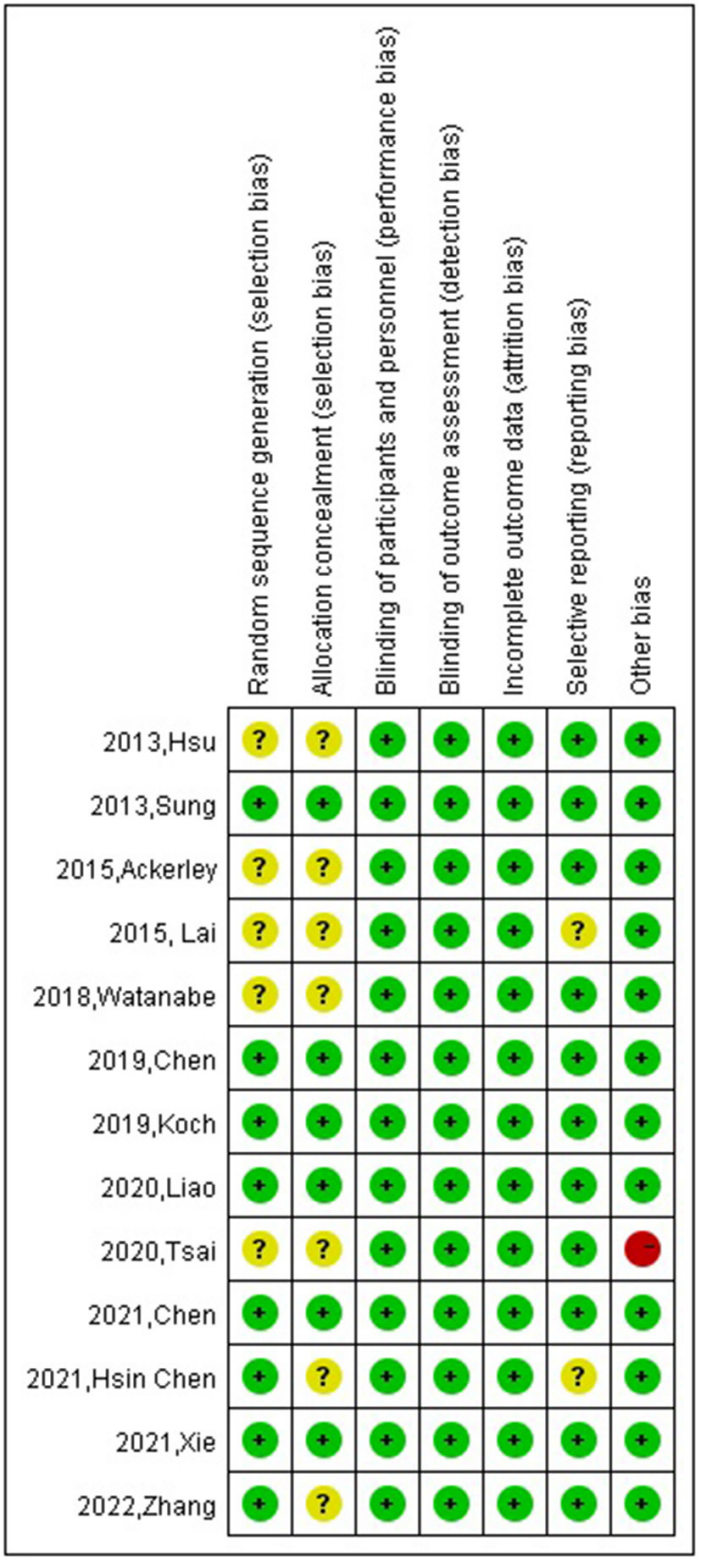
Risk of bias: a summary table for each risk of bias item for each study.

Figure 5 shows the details of the risk bias for all studies. Four trials had an unclear risk of bias in random sequence generation. For allocation concealment, the risk of bias was unclear in seven studies. For selective reporting, the risk of bias was unclear in two studies. Apart from these items, one trial had high other biases.

### PEDro scale of included studies

**Figure 6:**
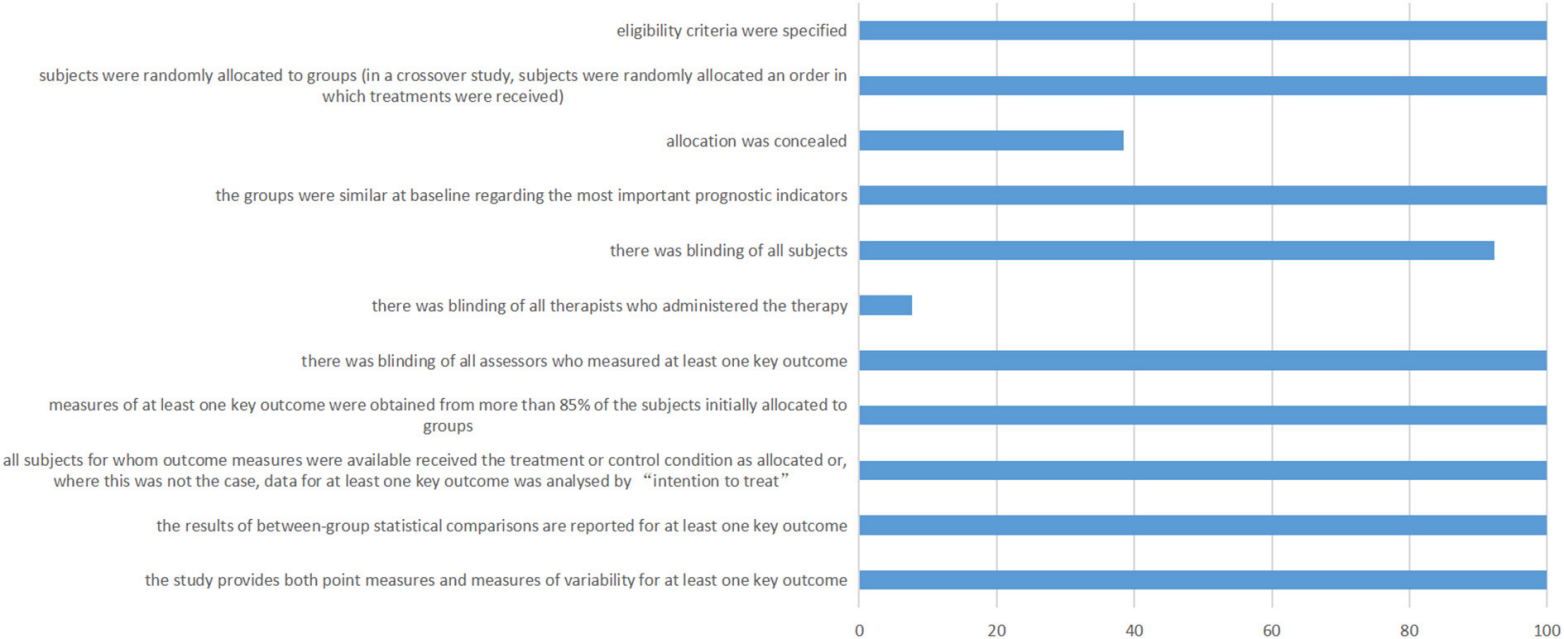
PEDro scale of included studies. The horizontal axis represents the percentage of studies.

Figure 6 shows the details of the Pedro scale of included studies. The allocation of eight studies was not concealed. One study was not blinding of all subjects. Twelve studies were not blinding of all therapists who administered the therapy.

## Discussion

Our meta-analysis included 13 RCTs containing 334 patients (iTBS = 170, Sham = 164), which provided high levels of clinical reliability in assessing the effect of iTBS on improving poststroke motor function. According to the results of our meta-analysis, iTBS could significantly improve the FMA score but not in MAS score. Moreover, it seems that standard 600-pulse stimulation of iTBS is more beneficial for motor function improvement than standard 1200-pulse stimulation. Nonetheless, the effect of iTBS for improving motor function only exists in chronic stroke patients but not in subacute patients. Besides, we also assessed other outcomes such as ARAT, WMFT, BBS, and BI. The results showed that iTBS could only improve motor function but not enough to improve balance and life quality after stroke.

When assessing the motor function improvement, we found that iTBS could only improve the FMA score but not the MAS score. This may be related to the fact that the FMA assessment focuses on coordinated and dissociative movements, while the MAS assessment focuses on items related to activities of daily living. This finding seems to indicate that the role of iTBS is mainly reflected in the coordinated and dissociative movements of the patient's limbs, and it has little effect on the improvement of life-related functions, which is also consistent with our analysis of the BI index that iTBS did not improve patients' quality of life. This interpretation may also help us understand why iTBS improves ARAT but not WMFT scores. Compared with ARAT, many complex actions involved in WMFT, such as stacking towels, and turning a key in the lock, are more akin to daily activities. However, there is no possible evidence to support that iTBS cannot improve balance function. The result may be due to the lack of included literature, and the effect of iTBS on balance function can be further studied in the future.

The results of our meta-analysis showed that the included studies were highly heterogeneous. We first suspected that the heterogeneity might be due to differences in the populations and methodologies of the included studies. Among the included studies, stroke progression and the iTBS regimen used varied, which may have affected the effect of iTBS on motor recovery after stroke. Therefore, sensitive and subgroup analyses were performed to explore the origin of heterogeneity from a statistical and methodological perspective. Sensitive analysis showed the pooled results were stabled when assessing the FMA, MAS, and ARAT. Moreover, the results of BBS and BI were not changed after excluding highly sensitive trials. The sensitive analysis revealed that the heterogeneity was not sourced from a single study. However, the effect of iTBS in improving FMA scores was unstable in different subgroups. Though iTBS could benefit both upper and lower extremity motor function, it could only benefit chronic stroke patients rather than subacute ones. Tscherpel's study showed that the resting motor threshold (RMT) of the cerebral cortex decreased 1 week to 3 months after stroke and then increased in the chronic phase (45). This result revealed that the excitability of the cerebral cortex was elevated in the acute and subacute phases and subsequently suppressed in the chronic phase. Another study showed that non-primed iTBS could increase the input-output curve of motor-evoked potentials (IO-MEP), which revealed that iTBS could increase cortical excitability (46). For the recovery-promoting effect of iTBS, increasing the excitability of suppressed cortex is the primary therapeutic mechanism. Therefore, the effect of iTBS on promoting motor function recovery in post-stroke patients only exists in the chronic phase. Nonetheless, this possibility still needs to be confirmed by related mechanism studies. The management of iTBS in most studies was standard 600-pulse stimulation, while in several trials, 1200-pulse stimulation was also used in post-stroke treatment. Briefly, 1200-pulse stimulation can seem like two sessions of standard 600-pulse stimulation with a 10–15 min break (42). However, 1200-pulse stimulation of iTBS could not benefit patients' recovery. Murakami's study showed that the pairing of identical protocols (iTBS → iTBS) resulted in the suppression of the non-primed TBS effects on IO-MEP. This result revealed that 1200-pulse iTBS produces less cortical excitatory than 600-pulse (24). Therefore, 1200-pulse iTBS or cTBS might be more suitable for acute or subacute stroke patients. However, due to the lack of monitoring of cortical excitability, related mechanism studies are still needed to confirm.

There are still several limitations in our studies except for those mentioned above. First, there is no subclassification of hemorrhagic and ischemic stroke patients. Therefore, results may vary due to different pathologic mechanisms in hemorrhagic and ischemic stroke patients. Second, although 13 RCTs were included, the sample size of patients included was still small, and larger RCTs are needed to further explain the role of iTBS. Third, as most of the adjuvant treatments in the included studies were routine physical therapy, it was difficult to compare the effects of different physical therapy on iTBS in promoting motor function recovery after stroke. Finally, we did not compare the therapeutic effect of iTBS and rTMS, so more studies are needed to elucidate further the therapeutic difference between the two non-invasive brain stimulation methods.

## Conclusion

This study supports that iTBS has good efficacy for improving motor function in stroke patients even though iTBS could not lead to a reduced MAS score. Standard 600-pulse stimulation is suitable for iTBS therapy, however, 1200-pulse iTBS showed no effect on motor function improvement after stroke. Besides, iTBS has a better effect on motor function improvement in chronic stroke patients than in subacute ones.

## Data availability statement

The original contributions presented in the study are included in the article/Supplementary material, further inquiries can be directed to the corresponding authors.

## Author contributions

Zhw is the principal investigator. BG and DZ designed the study and developed the analysis plan. BG and ZoW analyzed the data and performed meta-analysis. BG, DZ, and YW contributed in writing of the article. ZhW and ZoW revised the manuscript and polished the language. All authors contributed to the article and approved the submitted version.

## Funding

This work was supported by the Suzhou Health Talents Training Project (GSWS2019002), the National Natural Science Foundation of China under Grant [82002643], and China Postdoctoral Science Foundation under Grant [2019M651954].

## Conflict of interest

The authors declare that the research was conducted in the absence of any commercial or financial relationships that could be construed as a potential conflict of interest.

## Publisher's note

All claims expressed in this article are solely those of the authors and do not necessarily represent those of their affiliated organizations, or those of the publisher, the editors and the reviewers. Any product that may be evaluated in this article, or claim that may be made by its manufacturer, is not guaranteed or endorsed by the publisher.

## References

[1] Campbell BCV De Silva DA Macleod MR Coutts SB Schwamm LH Davis SM . Ischaemic stroke. Nat Rev Dis Prim. (2019) 5:70. 10.1038/s41572-019-0118-8 31601801

[2] D'Anci KE Uhl S Oristaglio J Sullivan N Tsou AY. Treatments for poststroke motor deficits and mood disorders: a systematic review for the 2019 us department of veterans affairs and us department of defense guidelines for stroke rehabilitation. Ann Intern Med. (2019) 171:906-+. 10.7326/M19-2414 31739315

[3] Langhorne P Bernhardt J Kwakkel G. Stroke care 2 Stroke rehabilitation. Lancet. (2011) 377:1693–702. 10.1016/S0140-6736(11)60325-5 21571152

[4] Liao X Xing G Guo Z Jin Y Tang Q He B . Repetitive transcranial magnetic stimulation as an alternative therapy for dysphagia after stroke: a systematic review and meta-analysis. Clin Rehabil. (2017) 31:289–98. 10.1177/0269215516644771 27113337

[5] Vabalaite B Petruseviciene L Savickas R Kubilius R Ignatavicius P Lendraitiene E. Effects of High-Frequency (HF) repetitive transcranial magnetic stimulation (rTMS) on upper extremity motor function in stroke patients: a systematic review. Medicina. (2021) 57:1215. 10.3390/medicina57111215 34833433 PMC8617907

[6] Krogh S Jonsson AB Aagaard P Kasch H. Efficacy of repetitive transcranial magnetic stimulation for improving lower limb function in individuals with neurological disorders: a systematic review and meta-analysis of randomized sham-controlled trials. J Rehabil Med. (2022) 54:jrm00256. 10.2340/jrm.v53.1097 34913062 PMC8862648

[7] Yang XY He Q Fang F. Transcranial direct current stimulation over the visual cortex facilitates awake consolidation of visual perceptual learning. Brain Stimul. (2022) 15:380–2. 10.1016/j.brs.2022.01.019 35123143

[8] Hassan U Pillen S Zrenner C Bergmann TO. The brain electrophysiological recording and STimulation (BEST) toolbox. Brain Stimul. (2022) 15:109–15. 10.1016/j.brs.2021.11.017 34826626

[9] Suppa A Huang YZ Funke K Ridding MC Cheeran B Di Lazzaro V . Ten Years of theta burst stimulation in humans: established knowledge, unknowns and prospects. Brain Stimul. (2016) 9:323–35. 10.1016/j.brs.2016.01.006 26947241

[10] Huang YZ Lu MK Antal A Classen J Nitsche M Ziemann U . Plasticity induced by non-invasive transcranial brain stimulation: a position paper. Clin Neurophysiol. (2017) 128:2318–29. 10.1016/j.clinph.2017.09.007 29040922

[11] Diekhoff-Krebs S Pool E-M Sarfeld A-S Rehme AK Eickhoff SB Fink GR . Interindividual differences in motor network connectivity and behavioral response to iTBS in stroke patients. Neuroimage Clin. (2017) 15:559–71. 10.1016/j.nicl.2017.06.006 28652969 PMC5476469

[12] Ziemann U Siebner HR. Modifying motor learning through gating and homeostatic metaplasticity. Brain Stimul. (2008) 1:60–6. 10.1016/j.brs.2007.08.003 20633369

[13] Krakauer JW. Motor learning: its relevance to stroke recovery and neurorehabilitation. Curr Opin Neurol. (2006) 19:84–90. 10.1097/01.wco.0000200544.29915.cc 16415682

[14] Dileone M Ammann C Catanzaro V Pagge C Piredda R Monje MHG . Home-based transcranial static magnetic field stimulation of the motor cortex for treating levodopa-induced dyskinesias in Parkinson's disease: a randomized controlled trial. Brain Stimul. (2022) 15:857–60. 10.1016/j.brs.2022.05.012 35609815

[15] Jin G Chen J Du J He L Qi L Wu D . Repetitive transcranial magnetic stimulation to treat benign epilepsy with centrotemporal spikes. Brain Stimul. (2022) 15:601–4. 10.1016/j.brs.2022.04.003 35427811

[16] Spitz NA Pace BD Ten Eyck P Trapp NT. Early improvement predicts clinical outcomes similarly in 10 Hz rTMS and iTBS therapy for depression. Front Psychiatry. (2022) 13. 10.3389/fpsyt.2022.863225 35633811 PMC9130587

[17] Tang Y Chen H Zhou Y Tan ML Xiong SL Li Y . Analgesic Effects of repetitive transcranial magnetic stimulation in patients with advanced non-small-cell lung cancer: a randomized, sham-controlled, pilot study. Front Oncol. (2022) 12:840855. 10.3389/fonc.2022.840855 35372024 PMC8969560

[18] Liu C Pan W Jia L Li L Zhang X Ren Y . Efficacy and safety of repetitive transcranial magnetic stimulation for peripartum depression: a meta-analysis of randomized controlled trials. Psychiatry Res. (2020) 294. 10.1016/j.psychres.2020.113543 33238223

[19] Chastan N Etard O Parain D Gerardin P Fouldrin G Derambure P . Repetitive transcranial magnetic stimulation for patients with functional paralysis: a randomized controlled study. Eur J Neurol. (2022) 29:1293–302. 10.1111/ene.15264 35098613

[20] Huang YZ Edwards MJ Rounis E Bhatia KP Rothwell JC. Theta burst stimulation of the human motor cortex. Neuron. (2005) 45:201–6. 10.1016/j.neuron.2004.12.033 15664172

[21] Koch G Franca M Mochizuki H Marconi B Caltagirone C Rothwell JC. Interactions between pairs of transcranial magnetic stimuli over the human left dorsal premotor cortex differ from those seen in primary motor cortex. J Physiol London. (2007) 578:551–62. 10.1113/jphysiol.2006.123562 17124263 PMC2075160

[22] Talelli P Greenwood RJ Rothwell JC. Exploring theta burst stimulation as an intervention to improve motor recovery in chronic stroke. Clin Neurophysiol. (2007) 118:333–42. 10.1016/j.clinph.2006.10.014 17166765

[23] Chung SW Rogasch NC Hoy KE Fitzgerald PB. The effect of single and repeated prefrontal intermittent theta burst stimulation on cortical reactivity and working memory. Brain Stimul. (2018) 11:566–74. 10.1016/j.brs.2018.01.002 29352668

[24] Murakami T Muller-Dahlhaus F Lu MK Ziemann U. Homeostatic metaplasticity of corticospinal excitatory and intracortical inhibitory neural circuits in human motor cortex. J Physiol. (2012) 590:5765–81. 10.1113/jphysiol.2012.238519 22930265 PMC3528990

[25] Szaflarski JP Nenert R Allendorfer JB Martin AN Amara AW Griffis JC . Intermittent theta burst stimulation tbs for treatment of chronic post-stroke aphasia: results of a pilot randomized, double-blind, sham-controlled trial. Med Sci Monit. (2021) 27:e931468. 10.12659/MSM.931468 34183640 PMC8254416

[26] Li W Wen Q Xie YH Hu AL Wu Q Wang YX. *Improvement of poststroke cognitive impairment by intermittent theta* bursts: A double-blind randomized controlled trial. Brain Behav. (2022) 12:e2569. 10.1002/brb3.2569 35484991 PMC9226849

[27] Rao J Li F Zhong L Wang J Peng Y Liu H . Bilateral cerebellar intermittent theta burst stimulation combined with swallowing speech therapy for dysphagia after stroke: a randomized, double-blind, sham-controlled, clinical trial. Neurorehabil Neural Repair. (2022) 36:437–48. 10.1177/15459683221092995 35574927

[28] Koch G Bonni S Casula EP Iosa M Paolucci S Pellicciari MC . Effect of cerebellar stimulation on gait and balance recovery in patients with hemiparetic stroke A randomized clinical trial. JAMA Neurol. (2019) 76:170–8. 10.1001/jamaneurol.2018.3639 30476999 PMC6439971

[29] Xu S Yang Q Chen M Deng P Zhuang R Sun Z . Capturing neuroplastic changes after iTBS in patients with post-stroke aphasia: a pilot fMRI study. Brain Sciences. (2021) 11:1451. 10.3390/brainsci11111451 34827450 PMC8615629

[30] Zhang L Xing G Fan Y Guo Z Chen H Mu Q. Short- and long-term effects of repetitive transcranial magnetic stimulation on upper limb motor function after stroke: a systematic review and meta-analysis. Clin Rehabil. (2017) 31:1137–53. 10.1177/0269215517692386 28786336

[31] Liberati A Altman DG Tetzlaff J Mulrow C Gotzsche PC Ioannidis JPA . The PRISMA statement for reporting systematic reviews and meta-analyses of studies that evaluate health care interventions: explanation and elaboration. Ann Intern Med. (2009) 151:W65–94. 10.7326/0003-4819-151-4-200908180-00136 19622512

[32] Zhang JJ Bai Z Fong KNK. Priming intermittent theta burst stimulation for hemiparetic upper limb after stroke: a randomized controlled trial. Stroke. (2022) 53:2171–81. 10.1161/STROKEAHA.121.037870 35317611

[33] Xie YJ Wei QC Chen Y Liao LY Li BJ Tan HX . Cerebellar theta burst stimulation on walking function in stroke patients: a randomized clinical trial. Front Neurosci. (2021) 15:688569. 10.3389/fnins.2021.688569 34764848 PMC8576464

[34] Lin LF Chang KH Huang YZ Lai CH Liou TH Lin YN. Simultaneous stimulation in bilateral leg motor areas with intermittent theta burst stimulation to improve functional performance after stroke: a feasibility pilot study. Eur J Phys Rehabil Med. (2019) 55:162–8. 10.23736/S1973-9087.18.05245-0 30156086

[35] Chen YJ Huang YZ Chen CY Chen CL Chen HC Wu CY . Intermittent theta burst stimulation enhances upper limb motor function in patients with chronic stroke: a pilot randomized controlled trial. BMC Neurol. (2019) 19:69. 10.1186/s12883-019-1302-x 31023258 PMC6485156

[36] Watanabe K Kudo Y Sugawara E Nakamizo T Amari K Takahashi K . Comparative study of ipsilesional and contralesional repetitive transcranial magnetic stimulations for acute infarction. J Neurol Sci. (2018) 384:10–4. 10.1016/j.jns.2017.11.001 29249365

[37] Ackerley SJ Byblow WD Barber PA MacDonald H McIntyre-Robinson A Stinear CM. Primed physical therapy enhances recovery of upper limb function in chronic stroke patients. Neurorehabil Neural Repair. (2016) 30:339–48. 10.1177/1545968315595285 26180053

[38] Hsu YF Huang YZ Lin YY Tang CW Liao KK Lee PL . Intermittent theta burst stimulation over ipsilesional primary motor cortex of subacute ischemic stroke patients: a pilot study. Brain Stimul. (2013) 6:166–74. 10.1016/j.brs.2012.04.007 22659021

[39] Sung WH Wang CP Chou CL Chen YC Chang YC Tsai PY. Efficacy of coupling inhibitory and facilitatory repetitive transcranial magnetic stimulation to enhance motor recovery in hemiplegic stroke patients. Stroke. (2013) 44:1375–82. 10.1161/STROKEAHA.111.000522 23532011

[40] Lai CJ Wang CP Tsai PY Chan RC Lin SH Lin FG . Corticospinal integrity and motor impairment predict outcomes after excitatory repetitive transcranial magnetic stimulation: a preliminary study. Arch Phys Med Rehabil. (2015) 96:69–75. 10.1016/j.apmr.2014.08.014 25218256

[41] Chen Y Wei QC Zhang MZ Xie YJ Liao LY Tan HX . Cerebellar intermittent theta-burst stimulation reduces upper limb spasticity after subacute stroke: a randomized controlled trial. Front Neural Circ. (2021) 15:655502. 10.3389/fncir.2021.655502 34776874 PMC8578104

[42] Chen YH Chen CL Huang YZ Chen HC Chen CY Wu CY . Augmented efficacy of intermittent theta burst stimulation on the virtual reality-based cycling training for upper limb function in patients with stroke: a double-blinded, randomized controlled trial. J Neuroeng Rehabil. (2021) 18:91. 10.1186/s12984-021-00885-5 34059090 PMC8166006

[43] Liao LY Xie YJ Chen Y Gao Q. Cerebellar theta-burst stimulation combined with physiotherapy in subacute and chronic stroke patients: a pilot randomized controlled trial. Neurorehabil Neural Repair. (2021) 35:23–32. 10.1177/1545968320971735 33166213

[44] Verhagen AP de Vet HC de Bie RA Kessels AG Boers M Bouter LM . The Delphi list: a criteria list for quality assessment of randomized clinical trials for conducting systematic reviews developed by Delphi consensus. J Clin Epidemiol. (1998) 51:1235–41. 10.1016/S0895-4356(98)00131-0 10086815

[45] Tscherpel C Dern S Hensel L Ziemann U Fink GR Grefkes C. Brain responsivity provides an individual readout for motor recovery after stroke. Brain. (2020) 143:1873–88. 10.1093/brain/awaa127 32375172 PMC7296846

[46] Gopalakrishna Iyer N Shaha AR. Current concepts in the management of primary hyperparathyroidism. Indian J Surg Oncol. (2010) 1:112–9. 10.1007/s13193-010-0023-9 22930625 PMC3421003

