# Supplementary material for: Intermittent theta-burst stimulation with physical exercise improves poststroke motor function: A systemic review and meta-analysis
Source: Front Neurol. 2022 Aug 30;13:964627. doi: 10.3389/fneur.2022.964627 (PMC9468864; doi:10.3389/fneur.2022.964627)
Supplement: Supplementary file 1 [file Data_Sheet_1.docx]

Supplementary Material

## Supplementary Figures


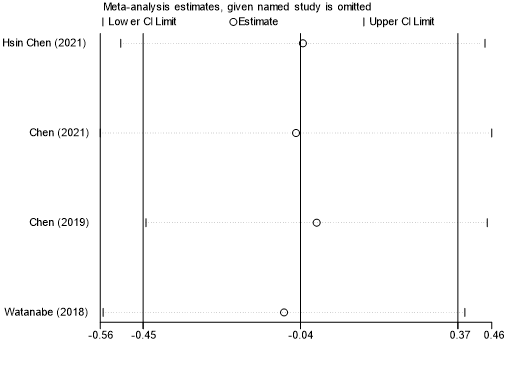


**Figure S1 Sensitive analysis of MAS.**


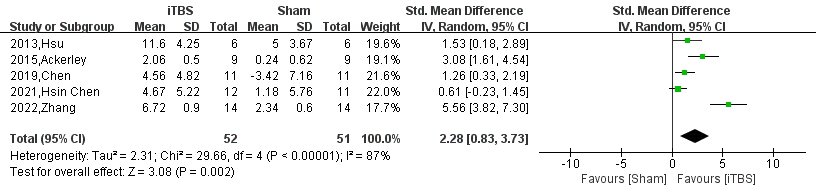


**Figure S2 Forest plot of ARAT**


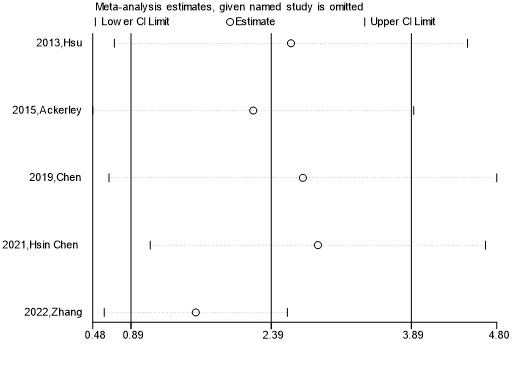


**Figure S3 Sensitive analysis of ARAT.**


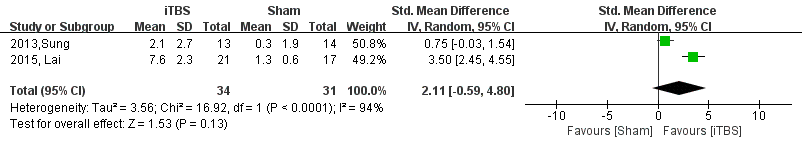


**Figure S4 Forest plot of WMFT**


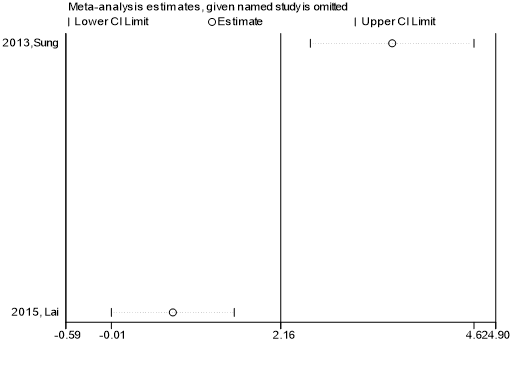


**Figure S5 Sensitive analysis of WMFT**


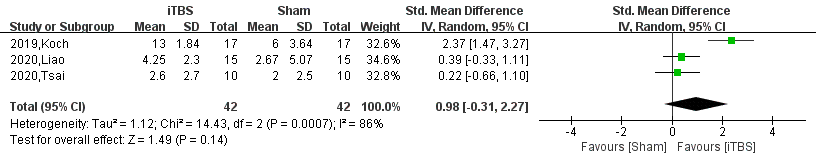


**Figure S6 Forest plot of BBS**


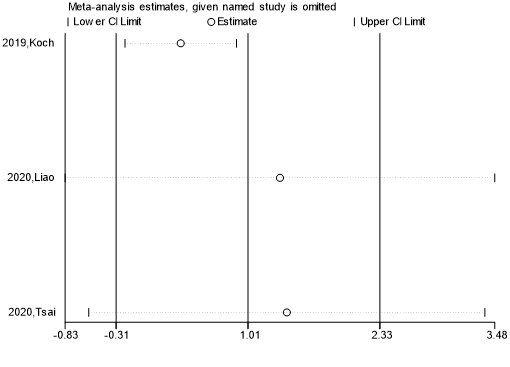


**Figure S7 Sensitive analysis of BBS**


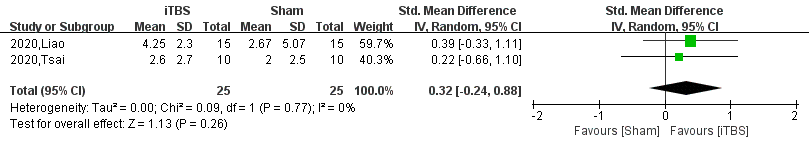


**Figure S8 Sensitive analysis of BBS after excluding the highly sensitive trial (2019, Koch)**


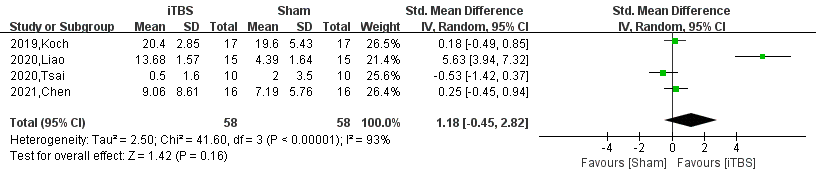


**Figure S9 Forest plot of BI**


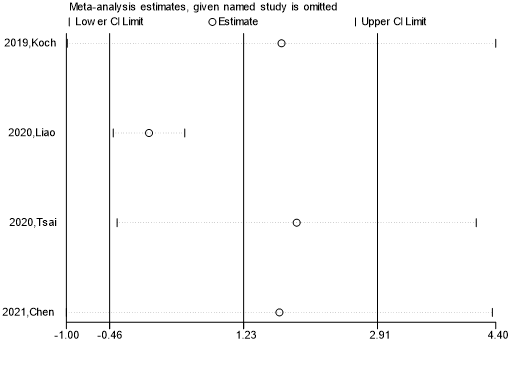


**Figure S10 Sensitive analysis of BI**


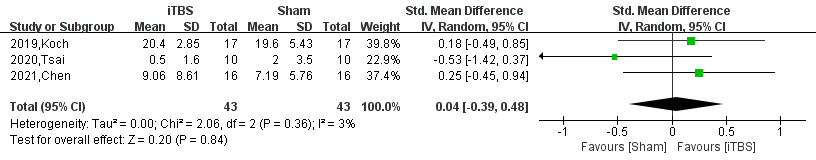


**Figure S11 Sensitive analysis of BI after excluding the highly sensitive trial (2020, Liao)**
